# School-Based Transdisciplinary Teaming to Maximize Behavioral Supports

**DOI:** 10.1007/s40617-025-01054-z

**Published:** 2025-04-14

**Authors:** Amber M. Reilly, Gabrielle E. Crowell, Jillian M. Thoele, Sarah DeAngelo, Moon Young Savana Bak

**Affiliations:** 1 Department of Educational Psychology, College of Education and Human Development, University of Minnesota, Room 250, 56 E. River Rd., Minneapolis, MN 55455 USA; 2 Department of Special Education, Peabody College, Vanderbilt University, Nashville, TN USA; 3 Department of Teaching and Learning, University of Iowa, Iowa City, IA USA; 4 Department of Special Education, University of Illinois Chicago, Chicago, IL USA

**Keywords:** Behavior analyst, Schools, Collaboration, Social validity, Ecological validity

## Abstract

In the United States school system, there is an increasing number of students who need behavioral support. Board Certified Behavior Analysts (BCBAs) utilize the science of applied behavior analysis and are uniquely qualified to provide such support. However, for many reasons, BCBAs may not have the knowledge and skills to capitalize on their services in schools, including collaboration skills that can result in goals, intervention procedures, and outcomes that are not socially and ecologically valid. The current article provides a transdisciplinary model of school-based collaboration for BCBAs to maximize the effectiveness of behavioral interventions in schools. Adapted for school-based BCBAs from Boyer and Thompson's (2014) transdisciplinary model, the current model includes the elements of (a) extending BCBAs’ knowledge regarding school systems, (b) establishing rapport with team members, (c) enriching team members’ understanding of everyone’s role and expectations, and (d) exchanging feedback and praise.

In the wake of the COVID- 19 pandemic, school-aged children worldwide have experienced an increased need for behavioral support, as is evidenced by increased reports of interfering behaviors (e.g., disruptions, disengagement, noncompliance; Musa & Dergaa, 2022). Even before COVID- 19, multiple studies in the extant literature have shown that students’ engagement in interfering behaviors can impact their ability to participate in instruction (Aldrup et al., 2018). For example, in their multivariate meta-analysis between student behavior and teacher burnout, Aloe et al. (2014) found students’ interfering behavior significantly affected teacher burnout. Interfering behaviors can put a strain on students’ relationships with their teachers and peers (Hirn & Scott, 2014; Musa & Dergaa, 2022) and increase their teachers’ stress and frustration (Hasting et al., 2002; Irwin et al., 2022; Nichols & Sosnowsky, 2002). Studies conducted post-COVID-19 discussed the increase in students’ interfering behavior and mental health difficulties, as well as their impact on teacher burnout (e.g., Herman et al., 2021; Oxley et al., 2024).

Issues stemming from teacher burnout and ineffective interventions are even more glaring in special education (Johnson & Jones, 2021). Special education requires effective individualized interventions for students based on scientifically sound evidence-based practices (EBPs) through the Individuals with Disabilities Education Improvement Act (Giangreco et al., 2023; IDEIA, 2004). Specifically, IDEIA (2004) enforces the use of EBPs in interventions and individualized education programs (IEPs). Many of these EBPs are developed on the basis of behavioral principles (e.g., positive reinforcement, functional behavior assessment; Barnett et al., 2020; Trump et al., 2018). However, research has shown that interventions are often implemented with poor fidelity in classroom settings (Hamrick et al., 2021; Hirsch et al., 2016; Max & Lambright, 2022), and in-service special educators continuously express the need for additional training to adapt EBPs for specific students in their classroom (Haspel & Lauderdale-Littin, 2020; Wang & Lam, 2017; West, 2018).

The inclusion of a Board Certified Behavior Analyst (BCBA) on school-based teams can mitigate many of the issues educators face involving feasibility, effectiveness, and fidelity of behavioral support implementation (Layden et al., 2024). For over 70 years, applied behavior analysis (ABA) has provided a systematic approach to developing, implementing, monitoring, and adapting instruction. Evidence for its utility in educational settings is rapidly increasing (e.g., Lory et al., 2024; Quigley et al., 2023; Runyon et al., 2018; Shepley & Grisham-Brown, 2019). As professionals who implement practices based on the science of ABA, BCBAs are uniquely positioned to provide support and training to teachers and other school-based professionals. As such, schools are increasingly employing BCBAs to address students’ need for behavioral support based on the effectiveness of ABA-based interventions across contexts (Layden et al., 2023; Shriver, 2019).

However, current BCBA training tends to focus more on a clinic-based model, and there are various ways in which BCBAs’ roles in the schools and clinics may differ (Layden, 2023). First, there are differences in the aim of their practice. For example, clinic-based BCBAs aim to manage or prevent overt interfering behaviors, whereas school-based BCBAs aim to increase skills that allow the student to access education in the least restrictive environment (Kuhn et al., 2020). Second, there are differences in the services they can provide in each setting based on systemic limitations, policies, and funding (Kuhn et al., 2020). Third, there are differences in the personnel they are responsible for working with. For example, in clinics, BCBAs commonly interact with individuals who likely understand the services provided by the BCBA (e.g., caregivers, behavior technicians, and other clinical professionals). Meanwhile, in schools, BCBAs often interact with a variety of interested parties, such as general education teachers, school psychologists, speech-language pathologists, and administrators.

As the benefits of ABA-based practices have been supported through empirical evidence throughout the years (refer to McCahill et al., 2014; Slane & Lieberman-Betz, 2021; included in Table 1), many school-based professionals receive training based on ABA principles (Barnett et al., 2020; LaFrance et al., 2019; Shriver, 2019). For example, as referenced in Table 1, Quigley et al. (2023) presented the foundational science behind ABA and how it can be used for positive behavior change in the general education classroom, and Lane et al. (2015) offered how school administrators understanding of positive behavior supports can provide effective school-wide interventions. Similarly, Fischer et al. (2021) and Lane and Brown (2023) discuss how school psychologists and speech-language pathologists, respectively, can integrate their training with behavior analysts and principles in ABA to enhance collaboration and practice for all professionals involved. However, the school-based professionals and practitioners BCBAs meet in their practice may have varying understandings of ABA (McCahill et al., 2014; McMahon et al., 2021; Menendez et al., 2017). As such, BCBAs need to understand the different roles and scope of practice of the school-based professionals for meaningful collaboration.

**Table 1:** Resources for BCBAs Working in Schools

Articles	Description
Bowman et al. (2021)	Standards for interprofessional collaboration in the treatment of individuals with autism
Broadhead (2018)	The Competence and Confidence Checklist
Critchfield (2014)	Rules for using behavior analysis to teach others about behavior analysis
DiGennaro Reed et al. (2019)	Guidelines for using behavioral skills training to provide teacher support
Kirkpatrick et al. (2019)	A review on the use of behavioral skills training with teachers
LaFrance et al. (2019)	Comparison and contrast of professions in behavior analysis, psychology, speech-language pathology, and occupational therapy
Lane et al. (2015)	Supporting behavior for school success: A step-by-step guide to key strategies
McCahill et al. (2014)	Training educational staff in functional behavioral assessment: A systematic review
Quigley et al. (2023)	Incorporating applied behavior analysis into the general education
Shriver (2019)	Applied behavior analysis in education: The role of the Board Certified Behavior Analyst
Schleeler et al. (2004)	A review on providing performance feedback to teachers
Slane and Lieberman-Betz (2021)	A review on using behavioral skills training to teach implementation of behavioral interventions to teachers and other professionals
Websites	Description
ERIC https://eric.ed.gov	Information related to various educational topics
Center on Positive Behavioral Interventions https://www.pbis.org	Information (including presentations and publications) as well as tools to implement Positive Behavioral Interventions and Supports
IRIS Center https://iris.peabody.vanderbilt.edu	Online evidence-based instructional and behavioral practices to support the education of students, particularly students with academic and/or behavioral challenges
National Center on Intensive Intervention https://intensiveintervention.org	Implementation guides, trainings, and tools to support the implementation of data-based individualization for students with severe and persistent learning and/or behavior needs
National Technical Assistance Center on Transition https://transitionta.org	Resources, guidance, and supports to aid teams in delivering effective services and education to secondary and post-secondary education students with disabilities
PROGRESS Center https://promotingprogress.org/	Resourcesand supports for educators to support the development of high-quality instructional programs for students with disabilities
TIES Center https://tiescenter.org	Tools to learn about inclusive education, including universal design for learning and positive behavior supports
What Works Clearinghouse https://ies.ed.gov/ncee/wwc/	Evaluations of products, practices, and policiesto determine their efficacy, aiding stakeholders in identifying effective interventions
Assessments	Description
Abbreviated Acceptability Rating Profile (Tarnowski & Simonian, 1992)	A shortened version of the Intervention Rating Profile- 15
Behavior Interventions Rating Scales (Von Brock & Elliot, 1987)	Six-point Likert-type scale used to assess intervention acceptability, effectiveness, and time of effectiveness
Children’s Intervention Rating Profile (Witt & Elliot, 1985)	A modified version of the Intervention Rating Profile- 15, written at a fifth-grade reading level
Intervention Rating Profile- 15 (Witt & Martens, 1983)	Six-point Likert-type scale used to obtain information on treatment acceptability from a teacher’s perspectives
Primary Intervention Rating Scale (Lane et al., 2009)	Adapted version of the Intervention Rating Profile- 15 to measure and monitor teacher’s opinions about school-wide programs
Structured Interview: Consumer Feedback on Treatment Decisions (Nicolson et al., 2020)	A set of questions developed to understand contextual factors that impact the intervention success
Treatment Acceptability Rating Form—Revised (Reimers & Wackers, 1988)	Seven-point Likert-type scale used to obtain information on treatment acceptability from the caregivers’ perspectives

Several resources were pulled from VanDerwall and Poling (2021).

There are also differences in the “clients” BCBAs serve. For example, in a clinic-based role, the BCBA might be responsible for providing individual services to a target child and their family (e.g., parent training); whereas in a school-based role, the BCBA might be responsible for providing services at the individual, classroom, and school levels for students with and without disabilities (VanDerwall & Poling, 2021). With a wide variety of roles and collaborators, a BCBA seeking to work within a school setting prioritizes acquiring the skills to provide individual, classroom, and school-level support and work collaboratively and harmoniously with other school-based professionals (Boivin et al., 2021; Bowman et al., 2021). Additionally, BCBAs are required to provide services that are within their scope of practice and competence (Brodhead et al., 2018), and it is important that BCBA trainees who want to work in schools receive the appropriate training to acquire specific skills needed for school settings (Layden, 2023; Light-Shriner et al., 2023).

As such, several studies have emphasized the need for and importance of school-specific training for BCBAs (see Fischer et al., 2021; Giangreco et al., 2023; Kelly & Tincani, 2013; Lalonde et al., 2022). Giangreco et al. (2023) recommend a collaborative teamwork framework to navigate the complex, ever-changing ecologies of schools that impact the services BCBAs provide and the roles they take on (Layden , 2023). Similarly, Lalonde et al. (2022) state the importance of interdisciplinary training for behavior analysts in training based on the diverse roles BCBAs may need to serve within a school context. These roles can span from working with a specific student on behavior reduction and skill acquisition, providing consultations to a teacher on classroom management strategies, to working with administrative personnel on the implementation of school-wide behavior supports (Max & Lambright, 2022).

The complexities of working with many other professionals on different tasks and roles require BCBAs to establish strong collaborative relationships with other school-based professionals (Bowman et al., 2021). Many disciplines in health and education discuss the importance of collaborative teams. However, terms such as interdisciplinary teams, multidisciplinary teams, and transdisciplinary teams are used interchangeably without understanding the differences between these different collaborative teaming methods (Choi & Pak, 2007).

In multidisciplinary teams, contributions are often additive and not integrative, which may result in siloed teams (Choi & Pak, 2006). Interdisciplinary teams preserve the discipline-specific roles of professionals but share goals and responsibilities (Choi & Pak, 2006). Transdisciplinary teams go beyond the traditional roles established within disciplines and capitalize on every member’s unique knowledge and skills to create socially and ecologically valid goals and interventions (Bowman et al., 2021). More specifically, the defining element of transdisciplinary teaming is shared roles in which members are trained to implement cross-disciplinary interventions (King et al., 2009). For instance, a BCBA may use behavioral skills training to teach a speech-language pathologist to use a token economy independently in their sessions. Intentionally planned transdisciplinary teaming, considered best practice within teaming frameworks, may be most effective for school-based BCBAs (Bowman et al., 2021).

A transdisciplinary teaming approach will facilitate the many roles a BCBA may have to serve within a school, such as consulting others; collecting and analyzing student-, classroom-, and school-level data; making data-based decisions; and providing direct training and coaching to other school-based professionals and relevant parties (Layden, 2023; VanDerwall & Poling, 2021). Therefore, the purpose of this article is to articulate a transdisciplinary model of school-based collaboration for BCBAs intended to maximize the effectiveness of behavioral interventions.

## A School-Based Transdisciplinary Model

**Fig. 1:**
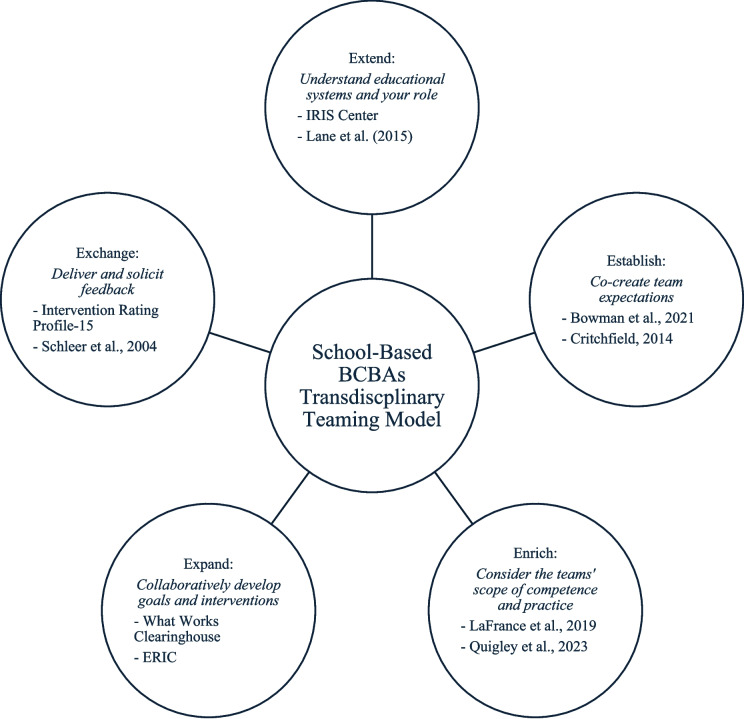
School-based BCBAs transdisciplinary teaming model

The recommendations provided in this article are adapted from Boyer and Thompson’s (2014) Transdisciplinary Model for Early Intervention and includes five elements – *extend, establish, enrich, expand,* and *exchange* – adapted for the school system. Refer to Fig. 1 for a visual with each element’s definition as well as aligned resources. After each element, a hypothetical case study will be provided, denoted by italics. For ease, resources to support school-based BCBAs are organized in Table 1.

## Extend: Understand Educational Systems

Extend, the first element of the transdisciplinary model, centers on a school-based BCBA’s understanding of their role and related educational laws and/or policies. For BCBAs at an introductory level, Shriver’s (2019) article on BCBAs in education and the website What Works Clearinghouse, which provides evidence on education programs, may be good starting points. ERIC is a website that BCBAs can use to search for a wide variety of information on educational topics. All are described in Table 1. School-based BCBAs will likely be impacted by their employment arrangement. BCBAs practice in schools through contractual arrangements with non-school agencies, or they may be hired by school districts directly (Giangreco et al., 2023). School-based BCBAs may need to participate in discussions requiring knowledge outside of clinic-based training, such as curriculum standards and academic assessments, to support their students. To collaborate effectively with a variety of team members, BCBAs seek knowledge related to their role within the school system. For example, if BCBAs are hired to support students with special education service eligibility, knowledge in relevant topics such as the IEP process, conducting FBAs in a school setting, and school-based disciplinary procedures are essential. Websites found in Table 1, such as the IRIS Center, the National Center on Intensive Intervention, the National Technical Assistance Center on Transition, and the PROGRESS Center, may be helpful for BCBAs serving students with special education services.

When hired by schools directly, the BCBA may be conceptualized as a related service provider under IDEIA 2004 (Giangreco et al., 2023). Under this distinction, a BCBA may be considered as a professional “required to assist a child with a disability to benefit from special education…” (IDEIA, 2004). Although IDEIA does not necessitate BCBAs be present at any point in the behavioral intervention process, it requires properly trained professionals to conduct FBAs and develop intervention plans. As BCBAs receive specialized training in analyzing the functions of behavior and developing aligned interventions, BCBAs can become the strongest source of support (VanDerwall & Poling, 2021). The IDEIA (2004) stipulation not only provides an avenue for BCBAs to consult in schools but also opens a source of funding, making their services in schools more ecologically valid (VanDerwall & Poling, 2021). It is recommended that BCBAs without an understanding of IDEIA (2004) undergo training to enhance their education instead of relying on learning from team members (Vladescu et al., 2022).

Whether employed as a related service provider within schools or an outside expert, school-based BCBAs will often participate in the IEP process for students with special education services (VanDerwall & Poling, 2021). A lack of understanding of procedural components can negatively impact a BCBA’s ability to apply their skillset (VanDerwall & Poling, 2021). For example, a BCBA may unknowingly recommend an intervention that conflicts with available school personnel, as they may be unaware of policies and norms surrounding this. Examples may include the use of sensory materials that are not readily available as an antecedent intervention within a certain school district or the use of a tablet device as a reinforcer in a school where device use is against school policy. However, BCBAs must also make best practice recommendations based on an individual student without prioritizing a school’s available resources. In particular, IDEIA (2004) emphasizes the inclusion of students with disabilities in the least restrictive environment (i.e., general education classrooms). As such, the BCBA’s ethical responsibility is to build their competence through education, training, and supervised experience to ensure they engage in optimal school-based services (Brodhead et al., 2018). As referred to in Table 1, Brodhead et al. (2018) developed the Competence and Confidence Checklist, a resource that can be used for BCBAs’ self-assessment.

BCBAs may benefit from increasing their familiarity with frameworks adopted by many schools, such as the multi-tiered systems of support (MTSS). MTSS is conceptualized as a three-tiered system in which the first tier is centered on universal and proactive evidence-based instruction, and the second and third tiers include more intensive academic (e.g., Response to Intervention; RTI) and behavioral supports (e.g., Positive Behavior Intervention and Support; PBIS; Clark & Dockweiler, 2020; Lane et al., 2015; Sugai et al., 2016). Students move among tiers contingent on their levels of academic and behavioral needs, determined through data-based decision-making and assessment (Sugai & Horner, 2009). BCBAs have the expertise to support students at any tier of PBIS based on their extensive training in data collection and analysis as well as intervention implementation. However, it is important that BCBAs appraise the match between intervention intensity and a student’s behavioral need(s) to ensure the provision of socially and ecologically valid interventions, as well as actively searching for training and learning opportunities to increase their understanding of the school and district systems (Horner et al., 2017). Three websites included in Table 1, the Center on Positive Behavioral Interventions, the IRIS Center, and the TIES Center, can be utilized by BCBAs to learn more about MTSS, RTI, and PBIS.

*Mrs. Wallace is a BCBA who has a wealth of experience providing ABA-based services in a clinical setting, but after a relocation, she started providing consultation services at a public school. First, she talks to the principal about her role, including whether she will support children with special education services. After learning that she will support students with and without disabilities, Mrs. Wallace consults IRIS modules to learn more about legal regulations and reaches out to the special education team leader, Mrs. Tidwell, for a meeting. Mrs. Wallace also asks to observe different settings throughout the school. Mrs. Wallace notes that teachers use AIMsweb for academic benchmarking and a “points system” to monitor the students’ behavioral needs. With this information, she sets up a meeting with an instructional coach, Dr. Lynberg, to learn more about academic standards and assessments as well as their alignment, if any, to MTSS. Mrs. Wallace then schedules a follow-up meeting with the principal to learn more about the “points system” and its relation to PBIS.*

## Establish: Co-Create Team Expectations

Establish, the second element of the transdisciplinary model, relies on the co-creation of team expectations to establish relationships that enable effective collaboration centered on student outcomes. Once a BCBA’s role in the school is established, the BCBA can identify and meet with school-based personnel with whom they are likely to collaborate. The introductory meeting can consist of the BCBA sharing information about the scope of their role and how they intend to operate. During an initial meeting, it may be appropriate for BCBAs to help school personnel understand the details of their role and address any possible misconceptions about ABA some practitioners may have (e.g., coercion, unwillingness to collaborate) using language free of jargon (Critchfield, 2014). As referred to in Table 1, BCBAs can consult Critchfield (2014) for recommendations regarding utilizing behavior analysis to teach others about behavior analysis.

Some important information for BCBAs to learn during these meetings may include each team member’s preferences on communication modalities (e.g., in-person, phone, text, email), the timing of feedback (e.g., immediate, during a break period, or after the school day), and the structure of feedback (e.g., constructive comments for efficiency or constructive comments and positive comments to recognize current skills). The BCBA may also want to model and suggest the practice of sharing team members'work and communication preferences during meeting, so this practice becomes a reciprocal process within team members leading to a stronger collaborative team. At the beginning of the relationship, BCBAs may be considered outsiders, especially if their presence is regarded as a signal for improvement (Cicoria, 2019). Guidelines to initiate a collaborative relationship include humbly acknowledging the strengths and limitations of one’s skillset, sharing appreciation of others’ expertise and contributions, and listening more than talking (Circoria, 2019).

Introductory meetings can also serve as a way for team members to get to know one another and establish a shared mission that centers students in decision-making. Prioritizing students early in this process lays the foundation for fostering rapport and collaboration among team members. Although this process may seem tedious, positive first encounters foster candid conversations and willing collaboration, positively impacting outcomes (Falletta-Cowden et al., 2023). Collaborative relationships promote mutual respect and a sense of shared values and purpose among team members (Hong & Reynolds-Keefer, 2013). How these relationships are built depends on the parties involved, as teams are dynamic, and members have individual preferences and needs.

Establishing norms that align with reciprocal partnership is an important prerequisite for an earnest, collaborative relationship. BCBAs should work with team members to establish communication norms to facilitate open, mutual feedback and decrease the likelihood of miscommunication. Included as a resource in Table 1, Bowman et al. (2021) emphasize the importance of open, ongoing, and active communication; sharing information; and using mutually understood language. They also recommend that each team member share their respective professional code of ethics and that the team members adopt the most conservative (i.e., strictest) rules to maintain team cohesion. BCBAs can share the Ethics Code for Behavior Analysts (2020) to encourage others to share their own ethics codes. Sharing information that guides other professionals can improve communication.

*Mrs. Wallace extended her knowledge by contacting reputable resources, observing, and engaging with diverse school staff. With a better grasp of her consultation role, Mrs. Wallace begins to establish her team. With the responsibility to support the fourth-grade teaching team, following multiple observations, Mrs. Wallace joins the teams’ weekly meetings. Wearing an Eagles’ t-shirt, the school’s mascot, Mrs. Wallace introduces herself, sharing her experiences and hobbies, working to build rapport. After inviting the other team members to do the same, Mrs. Wallace also shares that she is open to feedback and prefers it in an immediate and written manner. As the newest member of the team, Mrs. Wallace asks if collaborative norms have been set. For Mrs. Wallace, maintaining integrity and aligning it with professional ethics is an important norm for her own success. To approach this matter, Mrs. Wallace suggests adding a discussion regarding professional ethics and how they may be different or similar between organizations/roles as an agenda item in the next team meeting. When holding this discussion, Mrs. Wallace connects her ideals that align with the team members and school policies to emphasize commonalities.*

## Enrich: Consider the Teams’ Scope of Competence and Practice

Enrich, the third element of the transdisciplinary model focuses on considerations for each team member’s scope of competence and practice. To accomplish this, the BCBA works to understand and embrace the experience of others while evaluating their own scope of competence and practice. A transdisciplinary school-based team will always include a student’s general or special education teacher, as well as other school-based personnel, based on the specialized needs of each student. For example, a student who experiences co-occurring internalizing challenges (e.g., trauma, depression) may have a school psychologist or social worker on their team. Team members must understand their roles and responsibilities in relation to the team to work in a complementary manner where all members contribute toward a common goal (LaFrance et al., 2019). In addition to understanding their own role, team members can work to understand each other’s roles to effectively delineate responsibilities and identify how each member will contribute to the team’s goals (Bowman et al., 2021). Understanding other team members’ roles will increase the likelihood that each member’s expertise is capitalized upon and assist the BCBA in identifying where their skill set best fits within a given team. BCBAs can also learn about different school-based professionals’ objectives, philosophical underpinnings, scopes of practice, and applicable national and state licensing requirements (LaFrance et al., 2019). Included in Table 1, LaFrance et al. (2019) compared professions, including behavior analysis, psychology, speech-language pathology, and occupational therapy.

Further, team members will vary in their discipline-specific knowledge and experiences. Working within one’s scope of practice and competence promotes ethical practice and positive outcomes for students. One’s scope of practice is determined by the possession of a certain credential (e.g., license or certification), and one’s scope of competence consists of different skills that can be demonstrated accurately and reliably (Wise, 2008). Team members, including the BCBA, should reflect on their own scope of competence, only practice what they are proficient in, and seek additional training to expand their scope of competence.

BCBAs may find themselves working with school-based professionals who are unfamiliar with certain practices or interventions. In these cases, BCBAs can consider interventions requiring no to minimal training, including interventions that are already in place in the classroom or are familiar to the teacher and student to allow BCBAs to identify practices that, with small adaptations, can produce optimal outcomes for students (Chezan et al., 2022). Grounding interventions in these familiar practices may promote team members’ buy-in. Buy-in can also be fostered through *continued* conversations with teachers and other team members. When implementing a new intervention or practice is necessary, existing rapport will help the BCBA facilitate discussions regarding the positive impacts a new intervention could have for students and teachers.

To broaden the scope of competence for school-based professionals, BCBAs can share their expertise through professional development with other team members. When planning for professional development, it is important for BCBAs to consider all team members’ strengths and weaknesses. Some areas that BCBAs may consider for professional development include specific behavioral programs (e.g., communication training), teaching practices (e.g., prompting, chaining, and shaping), intervention fidelity (e.g., fidelity checklists), or general behavioral principles (Max & Lambright, 2022). Training may be most effective using a well-researched training method such as behavioral skills training (Miltenberger et al., 2017).

*Mrs. Wallace was asked to support a student with special education eligibility for autism who is included in the general education classroom for math. After observation and meeting with Mrs. Johnson, the student’s math teacher, Mrs. Wallace collected data that supported the student’s need for an intervention to support solving long division problems, which was aligned to the student’s math calculation goal on their IEP. During her observation, Mrs. Wallace noticed a poster on the wall with math facts and thought that a visual support may be well suited. After discussion, Mrs. Wallace recommended a task analysis, an EBP for students with autism. Mrs. Wallace related the task analysis to instructions for putting together furniture, and Mrs. Johnson quickly agreed that this was a feasible strategy and stated that she liked that it could be used with all students, too. Mrs. Wallace relied heavily on Mrs. Johnson’s expertise in math to ensure that the task analysis was accurate and aligned with the state math standards. Mrs. Johnson suggested that Mrs. Wallace teach a mini-lesson on task analysis to other teachers because of the evidence-base, ease, and effectiveness.*

## Expand: Collaboratively Develop Goals and Interventions

Expand, the fourth element of the transdisciplinary model relies on collaboratively developed goals and interventions. To accomplish this, the school-based BCBA develops socially and ecologically valid goals driven by direct and indirect data. Once teams are aware of each member’s scope of competence and practice, they must reach a consensus about shared goals and corresponding interventions for the student based on direct and indirect data collected by the BCBA and other team members. The collection of data to inform goals and intervention selection will likely require the BCBA to enter a teacher’s classroom or observe another professional during their practice. When doing so, BCBAs exercise respect for the school-based professional’s environment and instruction by entering at an ideal time predetermined and agreed upon by all parties, as well as being considerate of spontaneous changes that can often happen within a school setting (Circoria, 2019). Simultaneously, BCBAs must identify appropriate times and settings for data collection that will most accurately represent the student’s skills and needs, as accurate baseline data enables professionals to identify appropriate intervention goals (Gresham, 2004). As such, BCBAs can work with school-based professionals to prioritize times and settings that are optimal for accurate data collection, in addition to meeting logistical needs.

Some sources recommend that BCBAs address goals important to teachers to foster buy-in (Circoria, 2019). Though teacher buy-in is of key importance, providing ethical services requires BCBAs to identify goals that are important to students. Although it is important to promote buy-in, it is imperative that BCBAs carefully examine the social validity of intervention goals. BCBAs can recommend the team consider three fundamental questions related to social validity: (a) What should we change? (b) How should we change it? and (c) How will we know it was effective? (Gresham & Lopez, 1996, p. 205). When collaborating on goal setting with other providers, it is paramount that BCBAs seek to understand their colleagues’ ideas as they apply to behavior analysis and emphasize shared understandings (Giangreco et al., 2023).

Once a goal is developed, teams work together to identify interventions that target behaviors of concern. Conflicts may arise if other team members recommend a nonbehavioral intervention (Brodhead, 2015). To avoid hindering implementation, all team members must respond professionally and promptly to the conflict. Resolution tactics based on protocols that acknowledge the conflict, gain the perspectives of all involved, and aim for prompt resolution are critical (Bowman et al., 2021). When a nonbehavioral intervention is suggested, the first consideration by a BCBA is ethical practice. According to the ethics code, BCBAs are required to provide scientifically supported intervention. Educational laws are similar in that they require the use of evidence-based interventions (Every Student Succeeds Act, 2015) and scientifically based practices (IDEIA, 2004). However, quality indicators of methodological rigor and acceptable levels of effectiveness vary across fields (VanDerwall & Poling, 2021), and BCBAs must think critically about the evidence base of the recommended intervention. As such, a working knowledge of evidence-based interventions used in schools is needed. If the recommended intervention does not have empirical evidence, the BCBA can use educational laws and professional ethics codes (e.g., the BACB ethics code) to communicate against implementing a potentially harmful intervention.

Regardless of the intervention, it is critical that the student’s inclusion in their least restrictive environment is at the center of the decision-making process (Slim & Reuter-Yuill, 2021). Overall, interventions that take place in typical settings, during naturally occurring routines, with typical implementers are considered more feasible (Ledford et al., 2016). Factors such as training requirements, resource availability, and physical environment are important considerations. Including all team members in the decision-making process can lead to identifying interventions with increased potential for success (Falletta-Cowden & Lewon, 2023). Often, professionals dominate the IEP process, so advocating for strategies to include students and families is paramount (Bacon & Causton-Theoharis, 2013). This is especially important when considering the social significance of the goals, interventions, and outcomes, as meaningfulness should be determined by all interested parties (Falletta-Cowden & Lewon, 2023). A team that is representative of all interested parties will have a more holistic understanding of the student’s educational needs and the implementation context, leading to a feasible and effective intervention (Giangreco et al., 2023).

Before intervention implementation begins, it is recommended that the team develop a progress monitoring program, including who will monitor data, how frequently, and the steps for fading support from the BCBA to the natural interventionist (e.g., teacher; Traub et al., 2017). To maximize their services, BCBAs acknowledge the realities of the classroom and their impact on data collection for school-based professionals. Collaborating with team members to develop a simple and effective system can ensure accurate and continuous data collection. Accurate and continuous data can also be a powerful communication tool for team members (Circoria, 2019). Providing updates on the student’s progress and intervention adaptation allows team members to take ownership of the intervention. Sharing graphic displays of the data can prompt team members, including the student, to reflect on the significance of the outcomes to determine if success has been achieved (Falletta-Cowden & Lewon, 2023).

Decisions about intervention adaptations should be made based on student-level data, as well as feedback from team members, including the plan for the BCBA to fade support. Eventually, the goal of the BCBA is to transfer total implementation responsibility from the BCBA to the routine school-based professional. As such, the plan for fading support is an integral part of intervention planning. For example, the BCBA may first start by working with the student, directly modeling the intervention for the classroom teacher. Slowly, the BCBA may increase training for classroom teachers while concurrently decreasing the amount of time they spend working directly with the students (Traub et al., 2017). No matter the plan for fading support, the BCBA’s presence during the initial stages of the intervention ensures it is being implemented with fidelity and supports data collection.

*Mrs. Wallace knew it was essential to develop goals and interventions collaboratively. After collecting multiple sources of data, Mrs. Wallace met with Mrs. Johnson to discuss reasonable goals. Mrs. Johnson stated that her goal is for the student to solve 100% of the math facts given without any verbal protests from the student. Mrs. Wallace asked more about the verbal protests, as she had not observed instances of this behavior. Mrs. Wallace and Mrs. Johnson agreed on a time, ten minutes before the transition to recess, when Mrs. Johnson stated that the behavior was likely to occur. After her own data collection, Mrs. Wallace shared that she would like Mrs. Johnson to collect more data. Although initially Mrs. Johnson seemed reluctant to accept another task during her instruction, Mrs. Wallace collaborated with her to find a system that allowed for both feasibility and accuracy.*

## Exchange: Deliver and Solicit Feedback

Exchange, the fifth and final element of the transdisciplinary model, prioritizes feedback. Aligned to this element, school-based BCBAs establish a systematic approach for delivering feedback to interested parties, solicit feedback regarding their role, and incorporate the knowledge and abilities of team members to optimize outcomes. BCBAs who serve as coaches engage in a dynamic process wherein collaborative interactions are pivotal in enhancing school-based professionals’ implementation fidelity (Johnson et al., 2018). Across professions, it is widely accepted that performance feedback is imperative to skill development, refinement, and retention (Turner et al., 2016). More specifically, teachers have identified performance feedback as the most effective way to improve treatment fidelity (DiGennaro Reed et al., 2019; Strohmeier et al., 2014). Performance feedback can be provided in various ways (e.g., vocally, graphically, written) and may be conceptualized as positive or corrective (DiGennaro Reed & Codding, 2014; Turner et al., 2016). Positive feedback is provided to increase the likelihood that the school-based professional will continue to repeat their behavior. Alternatively, corrective feedback is provided to change or decrease a behavior that the school-based professional is engaging in and may include a review of the incorrect performance, followed by modeling and practice (Turner et al., 2016). Referenced in Table 1, BCBAs can consult DiGennaro Reed et al. (2019) and Kirkpatrick et al. (2019) for guidelines and recommendations on using behavioral skills training with teachers.

Although the utility of performance feedback is widely endorsed, the provision of performance feedback must be thoughtful and individualized. That is, the BCBA should carefully consider what feedback to give, how it will be delivered, and with consideration toward the recipient’s preferences. Although general recommendations exist around providing more positive than corrective feedback, a skilled and empathetic BCBA can use both forms to promote the school-based professional's motivation to engage in actions that increase treatment fidelity (Turner et al., 2016). A few common tips to follow when delivering corrective feedback are to (a) be objective, (b) provide feedback as soon as possible, (c) give suggestions for improvement in one area at a time, and (d) deliver corrective feedback in a private manner (Miltenberger, 2017; Scheeler et al., 2004; Turner et al., 2016). It is important to remember that the most effective feedback is based on the feedback recipient’s behaviors after initial feedback is given. Thus, BCBAs are systematic when providing feedback and collect data to analyze the impact, if any, of feedback on the school-based professional’s subsequent performances. By using data to gauge the effectiveness of their own feedback, BCBAs can take appropriate action to improve their skills in delivering high-quality, individualized feedback. Included in Table 1, Scheeler et al. (2004) completed a review on providing performance feedback to teachers, complete with implications and recommendations.

BCBAs receiving feedback regarding their own performance is equally important. Although BCBAs can actively solicit feedback from team members, some school-based professionals may be reluctant or unsure how to provide direct feedback based on school culture and their training (Drago-Severson & Blum-DeStefano, 2018). In such a case, BCBAs can solicit feedback by utilizing surveys such as rating scales and questionnaires (Carroll & St. Peter, 2014). There are a variety of rating scales available for assessing treatment acceptability; however, to ensure appropriateness, BCBAs should select one that applies to the correct setting (e.g., classroom) and team member (e.g., classroom teacher) who is acting as the respondent. Surveys can contain questions regarding an intervention’s social and ecological validity, such as the likelihood of continued treatment use, the acceptability of the intervention for the classroom setting, and the appropriateness of the intervention for the target student (Common & Lane, 2017). BCBAs can also supplement and further validate the survey outcomes by conducting brief follow-up interviews. Refer to the final section of Table 1 for a collection of assessments that BCBAs can utilize to receive feedback on their interventions.

Although team member feedback provides essential information about an intervention’s acceptability and continued use, student input is also as valuable. In addition to the indirect methods, BCBAs can use direct assessment methods, such as a concurrent chain arrangement (Hanley et al., 1997), to assess the likelihood that the students will select an intervention and participate in the desired context.

*Mrs. Wallace reported that they felt the collaboration between Mrs. Johnson and herself was quality, but she knows the importance of objective feedback. To solicit this, Mrs. Wallace asked Mrs. Johnson to complete the Primary Intervention Rating Scale. Mrs. Wallace also asked a paraprofessional who works closely with the student about his perspective on the intervention as well as the student's. During the student’s IEP meeting, the student shared that they like using the “instructions” because it is a way to remember the steps without constantly asking for help. However, Mrs. Johnson disagrees with the use of pictures for the instructions and wants to use text instead. After consideration of Mrs. Johnson’s comments, Mrs. Wallace suggests slowly fading the use of pictures to text with the student’s reading level in mind.*

## Conclusion

A school-based BCBA skilled in collaboration integrates the expertise of team members as well as their unique skills to maximize student outcomes. However, some BCBAs may not be equipped with the necessary skills to implement behavioral plans effectively within idiosyncratic classroom and school settings. Grounded in Boyer and Thompson's (2014) Transdisciplinary Model, we recommend that BCBAs working in schools (a) understand educational systems, (b) co-create team expectations, (c) consider the team’s scope of competence and practice, (d) collaboratively develop goals and interventions, and (f) deliver and solicit feedback. With quality collaboration supported by intentionally planned transdisciplinary teaming, students achieve higher academically, and teachers improve their own instruction (Ronfeldt et al., 2015). Substantial evidence exists on the negative relationship between academic achievement and interfering behavior, and there is evidence that collaborative problem-solving positively affects targeted student behavior (Allen et al., 2003; Kremer et al., 2016).

For optimal impact, BCBAs can conceptualize school-based personnel as collaborators with valuable skills and knowledge. Maximizing school-based behavior support also warrants actions within the field of ABA. It necessitates the establishment of professional networks specifically for school-based BCBAs, which focus on continuing education initiatives and an increase in relevant scholarship (Layden, 2023). Moreover, the field of ABA should move toward mandating pertinent training and practical experiences tailored for school environments to maximize the positive effects BCBAs can have in schools.
